# The neuropathology of kuru and variant Creutzfeldt–Jakob disease

**DOI:** 10.1098/rstb.2008.0086

**Published:** 2008-11-27

**Authors:** Catriona A. McLean

**Affiliations:** Department of Pathology, The Alfred HospitalCommercial Road, Prahran, Victoria 3181, Australia

**Keywords:** kuru, Creutzfeldt–Jakob disease, neuropathology

## Abstract

A comparison of the pathological profiles of two spongiform encephalopathies with a similar presumptive route of infection was performed. Archival kuru and recent variant Creutzfeldt–Jakob disease (vCJD) cases reveal distinct lesional differences, particularly with respect to prion protein, suggesting that the strain of agent is important in determining the phenotype. Genotype analysis of the polymorphism on codon 129 reveals (in conjunction with updated information from more kuru cases) that all three genotypes (VV, MV and MM (where M is methionine and V is valine)) are detected in kuru with some preference for MM homozygosity. The presence of valine does not therefore appear to determine peripheral selection of PrP^CJD^. vCJD remains restricted to date to MM homozygosity on codon 129. It remains to be determined whether this genotype is dictating a shorter incubation period.

McLean *et al*. (1998) reviewed 11 archival cases of kuru held in the University of Melbourne. A comparative study of the pathology and the host genotype, particularly the naturally occurring (‘public’) polymorphism on codon 129, was conducted using these kuru cases and 11 new variant Creutzfeldt–Jakob disease (vCJD) cases from the UK. The triad of spongiform change, neuronal loss and gliosis was seen in all kuru cases in a distribution similar to that originally described, with prominent involvement of all cortical areas with the exception of the occipital cortex, hippocampus and insular gyri, and prominent changes also seen in the putamen, caudate and cerebellar cortex (figure 1; Fowler & Robertson 1958; Klatzo *et al*. 1959; Neumann *et al*. 1964; Kakulas *et al*. 1967; Beck & Daniel 1979).

Neuropathological comparison of vCJD and kuru revealed that these diseases show distinct differences, particularly with PrP^CJD^ immunohistochemistry, where there was a much greater PrP load in all brain areas in vCJD than in kuru, with the exception of the cerebellar granular layer (table 1). The patterns of immunohistochemical changes in the brain also varied between vCJD and kuru (table 2). A comparison of the phenotype of kuru with more recent subtyping of Creutzfeldt–Jakob disease (CJD) shows that the kuru cases resemble those cases of sporadic CJD with type 2 PrP^CJD^ (Parchi *et al*. 1996). McLean *et al*. (1998) reported immunoperoxidase studies using the antibody 3F4 (SIGNET). A review of selected cases in 2008 using 12F10 (no. 189710 Cayman Chemical) highlights stronger and more pronounced reactivity (figure 2) than with the original 3F4 immunoperoxidase studies reported earlier.

The underlying differences in neuropathological profiles were thought to be in keeping with the different clinical features in these two disorders. Although both diseases exhibit prominent cerebellar ataxia, vCJD lacks the chronic emotional lability characteristic of kuru, and is characterized instead by psychiatric and sensory symptoms at onset with subsequent myoclonus and other movement disorders followed by dementia and akinetic mutism (Will *et al*. 1996).

The 11 cases of kuru showed a homogeneous pattern of prion protein deposition with an accentuation of cerebellar changes. Of the five cases in which the codon 129 could be assessed, there were two MM and three VV genotypes (M, methionine; V, valine; McLean *et al*. 1998). Analyses of codon 129 in kuru have shown that those patients with an MM genotype were preferentially affected and those with MV and VV genotypes appeared to be predisposed to a lower risk of disease development and longer incubation times (Cervenáková *et al*. 1998; Lee *et al*. 2001; see also Hainfellner *et al*. 1997; Lantos *et al*. 1997). Similarly, Collinge *et al*. (2006), in a study of 11 identified patients with kuru with assessabled incubation periods of 39–56 years, found they were heterozygous at the polymorphic codon 129 and believe this genotype is associated with an extended incubation period. The presence of MM homozygotes in kuru also suggests that it is not the presence of valine 129 that determines peripheral selection of the PrP^CJD^, as previously postulated to account for the high frequency of a valine 129 in other cases of iatrogenic CJD (Collinge *et al*. 1996). In vCJD, all cases to date have been MM homozygotes; however, vCJD has occurred within a very limited time frame compared with kuru (Cousens *et al*. 1997; Collinge *et al*. 2006). It therefore remains to be determined whether homozygosity at codon 129 dictates a shorter incubation period in vCJD. Moreover, the prolonged incubation period in kuru (over 50 years in some cases) may be significant when attempting to estimate future numbers of vCJD.

## Figures and Tables

**Figure 1:**
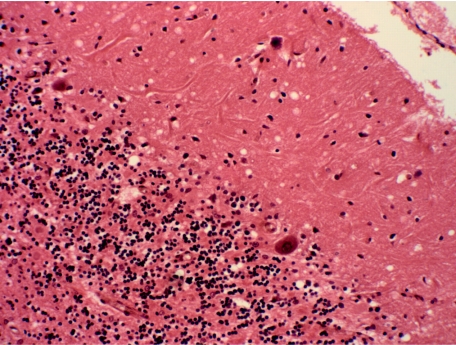
Kuru cerebellum showing spongiform change in the molecular layer, neuronal loss (Purkinje cells and granular cells) and gliosis (haematoxylin and eosin, ×200 actual magnification).

**Figure 2:**
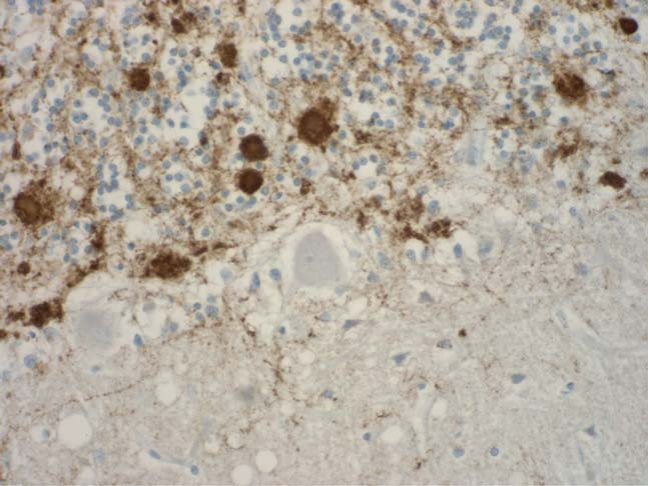
Kuru cerebellum showing strong immunoreaction with a granular pattern and plaque deposit in the granular layer and linear deposition within the molecular layer (12F10 prion protein, ×400 actual magnification).

**Table 1:** PrP^CJD^ load assessed on a scale of + to +++. (Comparison of the PrP^CJD^ load in vCJD and kuru by immunohistochemistry.)

PrP^CJD^ load	vCJD	kuru
frontal cortex	+++	+
basal ganglia	+++	+
thalamus	+++	+
cerebellar molecular layer	+++	+
cerebellar granular layer	+++	++–+++
basis pontis	++	+
spinal grey matter	++	+
substantia gelatinosa	+	+

**Table 2:** PrP^CJD^ patterns either present (+) or not present (−). (Comparison of the PrP^CJD^ patterns in vCJD and kuru by immunohistochemistry.)

PrP^CJD^ pattern	vCJD	kuru
florid plaques	+	−
linear/granular diffuse plaques	+	−
dendritic	+	+
perineuronal	+	+
cortical L3-5 laminar	−	+
accentuation		
linear white matter in the brain stem and the spinal cord	+	+
granular intracytoplasmic	+	−
